# IL-17A and TNF Modulate Normal Human Spinal Entheseal Bone and Soft Tissue Mesenchymal Stem Cell Osteogenesis, Adipogenesis, and Stromal Function

**DOI:** 10.3390/cells10020341

**Published:** 2021-02-06

**Authors:** Tobias Russell, Abdulla Watad, Charlie Bridgewood, Hannah Rowe, Almas Khan, Abhay Rao, Peter Loughenbury, Peter Millner, Robert Dunsmuir, Richard Cuthbert, Ala Altaie, Elena Jones, Dennis McGonagle

**Affiliations:** 1 Leeds Institute of Rheumatic and Musculoskeletal Medicine (LIRMM), University of Leeds, Leeds LS7 4SA, UK; umtjwr@leeds.ac.uk (T.R.); watad.abdulla@gmail.com (A.W.); C.D.Bridgewood@leeds.ac.uk (C.B.); umhmr@leeds.ac.uk (H.R.); medrjc@googlemail.com (R.C.); medaalt@leeds.ac.uk (A.A.); E.Jones@leeds.ac.uk (E.J.); 2 Zabludowicz Center for Autoimmune Diseases, Department of Medicine “B”, Sheba Medical Center, Tel-Hashomer, Ramat Gan 52621, Israel; 3 Sackler Faculty of Medicine, Tel Aviv University, Tel Aviv, Ramat Aviv 69978, Israel; 4 Leeds Teaching Hospitals NHS Trust, Leeds LS1 3EX, UK; almas.khan@nhs.net (A.K.); abhay.rao@nhs.net (A.R.); p.loughenbury@nhs.net (P.L.); Peter.millner@nhs.net (P.M.); robert.dunsmuir@nhs.net (R.D.)

**Keywords:** mesenchymal stem cells, enthesis, TNF, IL-17, spondyloarthritis

## Abstract

Objective: The spondylarthritides (SpA) are intimately linked to new bone formation and IL-17A and TNF pathways. We investigated spinal soft tissue and bone mesenchymal stem cell (MSC) responses to IL-17A and TNF, including their osteogenesis, adipogenesis, and stromal supportive function and ability to support lymphocyte recruitment. Methods: Normal spinal peri-entheseal bone (PEB) and entheseal soft tissue (EST) were characterized for MSCs by immunophenotypic, osteogenic, chondrogenic, and adipogenic differentiation criteria. Functional and gene transcriptomic analysis was carried out on undifferentiated, adipo- differentiated, and osteo-differentiated MSCs. The enthesis C-C Motif Chemokine Ligand 20-C-C Motif Chemokine Receptor 6 (CCL20-CCR6) axis was investigated at transcript and protein levels to ascertain whether entheseal MSCs influence local immune cell populations. Results: Cultured MSCs from both PEB and EST displayed a tri-lineage differentiation ability. EST MSCs exhibited 4.9-fold greater adipogenesis (*p* < 0.001) and a 3-fold lower osteogenic capacity (*p* < 0.05). IL-17A induced greater osteogenesis in PEB MSCs compared to EST MSCs. IL-17A suppressed adipogenic differentiation, with a significant decrease in fatty acid-binding protein 4 (*FABP4*), peroxisome proliferator-activated receptor gamma (*PPARγ*), Cell Death Inducing DFFA Like Effector C (*CIDEC*), and Perilipin-1 (*PLIN1*). IL-17A significantly increased the *CCL20* transcript (*p* < 0.01) and protein expression (*p* < 0.001) in MSCs supporting a role in type 17 lymphocyte recruitment. Conclusions: Normal spinal enthesis harbors resident MSCs with different in vitro functionalities in bone and soft tissue, especially in response to IL-17A, which enhanced osteogenesis and CCL20 production and reduced adipogenesis compared to unstimulated MSCs. This MSC-stromal-enthesis immune system may be a hitherto unappreciated mechanism of “fine tuning” tissue repair responses at the enthesis in health and could be relevant for SpA understanding.

## 1. Introduction

Ankylosing spondylitis (AS) is the paradigmatic seronegative spondyloarthritis (SpA) that is associated with axial inflammation and subsequent new bone formation that may progress to spinal fusion. Beyond AS, the normal aged spine shows a propensity for new bone formation in diffuse idiopathic skeletal hyperostosis (DISH) or degenerative arthritis, or as an incidental radiographic discovery [1,2]. Spinal new bone formation most characteristically occurs at the numerous entheseal locations and is thought to be linked to abnormal responses to stress, inflammation, or growth factors [3,4,5,6,7].

Multipotential stromal cells, also known as mesenchymal stem cells (MSCs), contain the osteoprogenitor pool that is thought to drive spinal bone formation [8]. Bone and periosteum-resident MSCs are known to play important roles in healthy skeletal tissue repair after injury, migrate to fracture sites, and differentiate into chondrocytes and osteoblastic cells of the fracture callus [9], as well as adipocytes in the long-bone marrow cavities of the elderly [10]. It is also known that adipocytes are present at the normal enthesis and may play an important biological role [7]. Moreover, in AS, the intraosseous anchorage sites of the annulus fibrosis are associated with post inflammatory adipogenic differentiation, which is a harbinger of future new bone formation in the adjacent anterior longitudinal ligament, but the mechanisms of this are poorly understood [11]. Other studies on AS have produced evidence for osteoprogenitors from the facet joints, but whether such cells are derived from bone or entheseal soft tissue has not been clearly defined and it has not been confirmed whether such cells are multipotential and hence represent MSCs [12].

Both tumour necrosis factor (TNF) and interleukin (IL)-17A are critically involved in AS pathogenesis, with the pharmacological antagonism of TNF eventually reducing new bone formation [13,14], whilst long-term results are awaited for IL-17A inhibition. Additionally, human iliac crest MSCs and MSCs from murine femurs display enhanced osteogenic differentiation after exposure to either IL-17A or TNF [15,16], supporting the rationale for IL-17A being potentially involved in aberrant bone reactions in AS. Normal murine and human spinal entheses also have tissue resident innate and adaptive immune cells capable of either IL-17A and TNF production, depending on the system and conditions, which points towards the proximity of immune cells and cytokines that could enhance or fine tune tissue repair responses for neighboring MSCs [17,18,19,20]. Indeed, stromal cells from AS patients and a healthy facet capsule reported enhanced osteogenic potential of the AS patient-derived cells, with IL-17A also driving increased osteogenesis from these same cells [12]. However, no study has specifically investigated differences in human enthesis ligamentous versus peri-entheseal bone MSCs, which is important, as new bone formation occurs in cells at the soft tissue entheseal location.

In addition to the fact that MSCs are the parent cells for osteoblasts, chondrocytes, and adipocytes, they are also known to be immunomodulatory via the secretion of immune cell mediators, including chemoattractants [21], notably C-C Motif Chemokine Ligand 20 (CCL20), which is a known T-cell chemoattractant via its interaction with C-C Motif Chemokine Receptor 6 (CCR6) [22]. Importantly, previous studies have identified a range of immune cell populations at the human spinal enthesis, which play a role in AS pathogenesis [18,20,23]; notably, populations of CD4+ and CD8+ T-cells capable of secreting both IL-17A and TNF [20]. With CCR6+ T-cells migrating towards CCL20 [22], the characterization of resident CCR6+ T-cell populations at the human spinal enthesis would help with the understanding of tissue repair responses exhibited by MSCs.

The aim of this study was to investigate MSC populations from the human spinal enthesis comprised of the peri-entheseal bone (PEB) and peri-entheseal soft tissue (EST) and to examine their responses to TNF and IL-17A, which are key disease-relevant cytokines [24], under osteogenic and adipogenic differentiation stimuli. Our findings show distinct functional properties of MSCs from these two sites and provide insights into how SpA-associated cytokines impact MSC biology, which in turn demonstrates how local MSC populations could be primed for aberrant tissue repair responses.

## 2. Materials and Methods

### 2.1. Participants and Samples

The investigation was approved by the North West-Greater Manchester West Research Ethics Committee (REC: 16/NW/0797). Patients gave informed consent, in accordance with the Declaration of Helsinki. Human PEB from the spinous process and EST from the interspinous ligament were collected from the normal spinous process and interspinous ligament of 38 patients (17 male, 21 female, median age 65, full sample list in Supplementary Table S3) undergoing spinal surgery at Leeds General Infirmary for the correction of scoliosis or spinal decompression of thoracic or lumbar vertebrae [18,19]. Entheseal tissue donors were not known to have any systemic inflammatory condition, including SpA. Tissue samples were dissociated from one another into PEB and EST using a scalpel. PEB was further broken down into <5 mm^2^ fragments by bone cutting forceps and EST was minced into a similar size using a scalpel before subsequent digestion with a collagenase solution, as previously described [25], with plating at 15,000 cells/cm^2^ in plastic tissue culture flasks and culture expanded in StemMACS MSC Expansion Media (Miltenyi Biotec, Bergisch Gladbach, Germany) and 1% penicillin-streptomycin until passage 2.

### 2.2. Cytokine Stimulations

IL-17A (50 ng/mL) and TNF (1 ng/mL) (both from PeproTech, Rocky Hill, NJ, USA) were employed for all differentiations in this study. These were used in concentrations similar to previous works looking at cytokine influences on osteogenesis in MSCs [26,27,28]. However, it is recognized that in vivo cytokine concentrations are much lower, usually in the pg/mL range [12,29], and the effects are mimicked using the concentrations employed in this and other studies. Combinations of the two were used in the same concentrations to investigate any synergistic effects between the two on osteogenesis or adipogenesis.

### 2.3. Colony Forming Unit Fibroblast (CFU-F) Assay for MSC Enumeration

The CFU-F potential of both PEB and EST was assessed after digestion by seeding 10,000 viable nucleated cells in 10 cm^2^ plastic Petri dishes containing 15 mL of StemMACS MSC expansion media and antibiotics. In duplicate, for each sample, cells were cultured for 14 days, with half media changes every 3 days. Cultures were fixed using 3.7% Formaldehyde and stained using a Methylene Blue solution [30,31]. Dishes were scanned at a 1200 dpi resolution and colonies were manually counted using ImageJ software v1.8.0_112 (U. S. National Institutes of Health, Bethesda, MD, USA); colonies were only counted if more than 50 cells were present. The percentage of colony forming cells from each digest was worked out using Equation (1):

(1) $$\%\mathrm{MSCs}=\;\frac{\mathrm{Number}\;\mathrm{of}\;\mathrm{Colonies}}{10000}\;\times \;100.$$

To investigate the individual colony area differences between PEB and EST MSCs, plates with more than 20 colonies had the area covered by each individual colony measured using ImageJ software v1.8.0_112 [32].

### 2.4. Flow Cytometry for Culture Expanded MSC Characterization

Passage 1 cultured cells from both PEB and EST were used to investigate surface marker expression according to the International Society for Cellular Therapy (ISCT) criteria for MSCs [33]. Prior to the addition of antibodies, cells were incubated with a blocking buffer (10% mouse serum and 1% human IgG in PBS). All incubations were conducted at room temperature for 15 min. Data was acquired using a Cytoflex S (Beckham Coulter, High Wycombe, UK) flow cytometer and analysed using FlowJo software V.10 (BD). MSCs were defined as being CD73/CD90/CD105^+^ and CD45/CD14/CD19/CD34/HLA-DR^-^ (Supplementary Table S1).

### 2.5. MSC Tri-Lineage Differentiation and Cytokine Stimulation

Minimally passaged (<p3) PEB and EST MSCs were assessed for their tri-lineage potential, as stipulated by the ISCT [33]. Briefly, for chondrogenesis, 250,000 cells (in triplicate for each sample) were pelleted and cultured in a chondrogenic media (CM) containing high glucose DMEM (ThermoFisher Scientific, Waltham, MA, USA), antibiotics, 40 μg/mL L-proline, 1.5 mg/mL bovine serum albumin, 4.7 μg/mL linoleic acid, 1× insulin–transferrin–selenium, 50 μg/mL L-ascorbic acid-2-phosphate, 100 nM dexamethasone (all from Sigma), and 10 ng/mL TGF-β3 (R&D Systems, Abingdon, UK) for 3 weeks. Half media changes were performed three times a week. The glycosaminoglycan (GAG) content of the pellets was assessed on the 21st day of differentiation using the Blyscan™ Glycosaminoglycan Assay (Bicolor, Carrickfergus, UK) [34].

For adipogenesis, 50,000 cells/well (in triplicate for each sample) were seeded in 24-well plates and cultured in complete adipogenic media (AM) containing low glucose DMEM (ThermoFisher Scientific, Waltham, MA, USA) supplemented with 10% foetal calf serum (FCS), 10% horse serum (Stem Cell Technologies, Sheffield, UK), 0.5 mM isobutylmethylxanthine, 60 μM indomethacin, 0.5 mM hydrocortisone (all from Sigma-Aldrich, Gillingham, UK), and antibiotics [35]. Half media changes were performed two times per week. At day 21, cultures were fixed using 10% formalin and stained with 0.5% Oil Red O (Sigma-Aldrich, Gillingham, UK) in isopropanol. Wells were imaged using an Olympus CKX41 light microscope equipped with an Olympus C-7070 camera, and images were analysed for the Oil Red O stained area using ImageJ Software v1.8.0_112 [35]. RNA extractions (Norgen, Thorold, Canada,) were performed for wells supplemented with IL-17A at day 0 on undifferentiated MSCs and at days 3, 7, 15, and 21.

For osteogenic cultures intended for alizarin red or alkaline phosphatase staining, 10,000 cells/well were seeded in 12-well plates (in triplicate for each sample) and cultured in complete osteogenic media (OM) containing low glucose DMEM supplemented with 10% FCS, 100 nM dexamethasone, 10 mM β-glycerophosphate, 0.05 mM ascorbic acid (all from Sigma-Aldrich, Gillingham, UK), and antibiotics [30]. Alkaline phosphatase (Sigma-Aldrich, Gillingham, UK) and alizarin red (Sigma-Aldrich, Gillingham, UK) staining was performed on days 14 and 21, respectively [36]. Stained wells were scanned at a 1200 dpi resolution using an Epson 3590 flatbed scanner.

Calcium quantification was used to assess potential changes induced by either IL-17A or TNF (in triplicate for each sample). For this, 3000 cells/well were seeded in 48-well plates and cultured for 14 days in complete osteogenic media [30] with either 5% or 10% FCS. At day 14, cultures were terminated and calcium was extracted by incubation with 600 mM hydrochloric acid for 4 h at 4 °C. Calcium levels were measured using a Calcium Liquid Colorimetric Assay Kit (Sentinel Diagnostics, Milano, Italy) [36].

### 2.6. Transcript Analysis

A total of 2 μL of RNA was reverse transcribed into cDNA using RT reagent (Fluidigm, San Francisco, CA, USA), using a programmed thermal cycler (Applied Biosystems, Foster City, CA, USA) under the following conditions: 5 min at 25 °C; 30 min at 42 °C; 5 min at 85 °C; and finally held at 4 °C. Pre-amplification of TaqMan probes (all ThermoFisher Scientific Waltham, MA, USA, Supplementary Table S2) and cDNA was carried out as previously described [32]. Transcript analysis was performed by qPCR using the Biomark HD gene expression system (Fluidigm, San Francisco, CA, USA), as previously described [18]. Hierarchal clustering of the data was performed using Cluster 3.0 and Java TreeView v.1.1.6 [31]. Genes showing a high degree of clustering were further analysed by normalizing values to the respective samples’ own undifferentiated MSCs using the ∆∆Ct method [37]. The use of this methodology allowed for patient variability to be accounted for in the downstream statistical analysis by assisting in the normalizing of values.

### 2.7. CCL20 Protein ELISA

Culture expanded MSCs from both PEB and EST (*n* = 6) were plated and allowed to expand to confluence in a 96-well plate, and they were subsequently stimulated with either IL-17A (100 ng/mL), TNF (10 ng/mL), or IL-17A and TNF for 48 h. These concentrations were selected due to other studies investigating the immunomodulation of MSCs using either IL-17A or TNF [38,39].

CCL20 levels in the supernatant were measured by ELISA (Biolegend, San Diego, CA, USA), as per the manufacturer’s instruction. Findings were correlated with stromal function analysis from transcript data, as described above.

### 2.8. CCR6 Expression

Enthesis samples were divided into PEB and EST and digested as before. Digested cells were incubated with 1% IgG and 10% mouse serum and subsequently stained for CD45+, CD3+ CD4+, CD8+, and CCR6 and analysed using the LSR II flow cytometer and FACS Diva software (BD Biosciences, San Jose, CA, USA).

### 2.9. Quantitative Real Time PCR for Transcript Analysis

T-cells were sorted using an Influx (BD Biosciences, San Jose, CA, USA) cell sorter directly into RNA extraction buffer, supplied as a component of the PicoPure RNA isolation kit (Thermofisher Scientific, Waltham, MA, USA). cDNA was synthesized with a reverse transcription kit (Fluidigm, San Francisco, CA, USA) and then underwent pre-amplification (18 cycles) using a pre-amp master mix (Fluidigm, San Francisco, CA, USA) with a solution containing all primer sets. All primers were purchased from Applied Biosystems. Transcript analysis was performed by qPCR using the Biomark HD gene expression system (Fluidigm, San Francisco, CA, USA), and values displayed are the log 10ΔCt relative to the Hypoxanthine Phosphoribosyltransferase 1 (*HPRT1*) housekeeping gene (Supplementary Table S2).

### 2.10. Statistical Analysis

Statistical analysis was performed using the IBM SPSS Statistics 24 software package v24.0.0.2. All data were assessed for normality using Shapiro–Wilks tests; this determined the statistical tests used to investigate the significance. The cytokine influence on adipogenesis and osteogenesis was tested using either repeated measures (RM) one-way ANOVA with a Tukey Post-Hoc comparison for parametric data or Friedman’s test with Dunn’s multiple comparisons for non-parametric data. Genes that showed high degrees of clustering after hierarchical clustering were subsequently analysed using paired *t*-tests. CCL20 protein ELISA was analysed using Friedman’s test with Dunn’s multiple comparison post-hoc test to compare to unstimulated controls. The Mann–Whitney U test was used to compare individual colony area and CCL20 protein production by ELISA.

## 3. Results

### 3.1. Numerical and Functional Characterization of PEB- and EST-Derived MSCs

**Figure 1:**
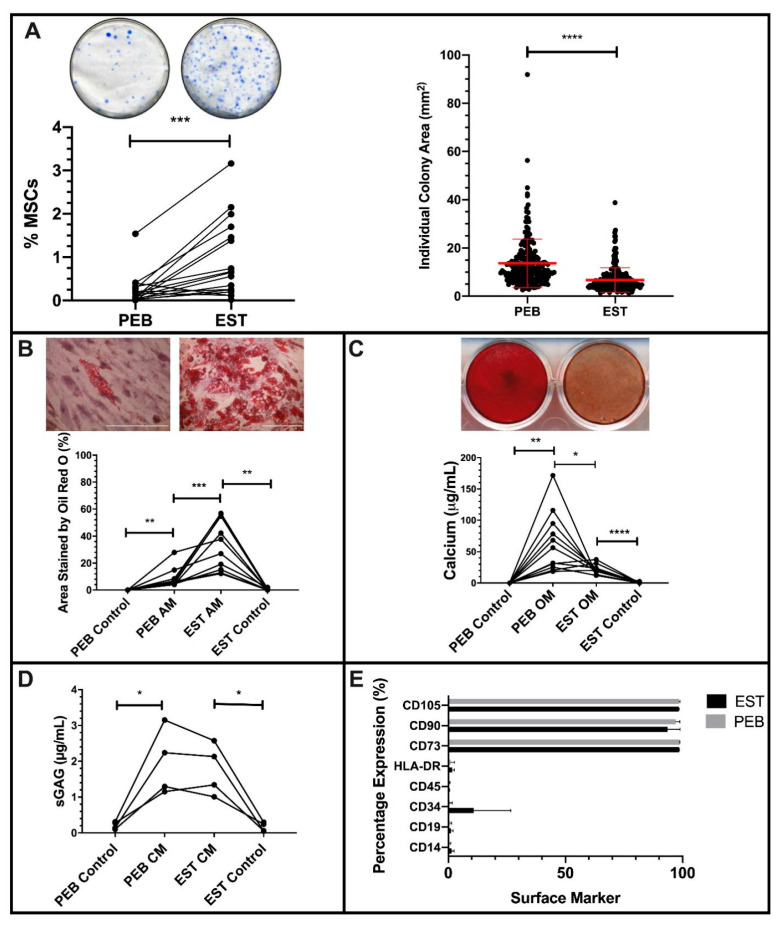
Characterization of peri-entheseal bone (PEB) and entheseal soft tissue (EST) mesenchymal stem cells (MSCs). Colony Forming Unit Fibroblast (CFU-F) ((**A**), Paired *t*-Test, *n* = 16) of PEB and EST MSCs shows a significantly higher CFU-F capacity of EST compared to PEB. Individual colonies are also significantly larger in PEB MSCs compared to EST MSCs (Mann–Whitney U Test). ESTs produce significantly more adipocytes than matched PEB MSCs after Oil Red O staining ((**B**), repeated measures (RM) One-Way ANOVA with Tukey Post-Hoc Test, *n* = 10). Alizarin red staining ((**C**), RM One-Way ANOVA with Tukey Post-Hoc Test, *n* = 11) of osteogenically differentiated PEB and EST MSCs show that matched PEB MSCs produce significantly more calcium than matched EST MSCs. There was no significant difference in the chondrogenic differentiation between PEB and EST MSCs ((**D**), RM One-Way ANOVA with Tukey Post-Hoc Test, *n* = 4). Both culture expanded PEB and EST MSCs (*n* = 3) were positive for MSC lineage markers and were negative for the hematopoietic lineage markers (**E**). Error bars = mean ± SD * = *p* < 0.05, ** = *p* < 0.01, *** = *p* < 0.001 and **** = *p* < 0.0001. Scale bar = 100 µm. AM = adipogenic media, CM = chondrogenic media, and OM = osteogenic media.

After sample digestion, EST digests contained 7.7-fold more (*p* < 0.0001) colony forming cells compared to donor matched PEB digests. This equated to 0.6% of viable nucleated cells compared to 0.12% in PEB (Figure 1A). Colonies of MSCs from PEB digests were significantly larger (*p* < 0.001) and more varied in diameter compared to EST digests, which were more similar in size (Figure 1A).

Both populations exhibited tri-lineage differentiation following culture in adipogenic, osteogenic, and chondrogenic media (Figure 1B–D). However, in adipogenic conditions, EST-derived MSCs had a significantly higher level of (*p* < 0.001) adipogenesis than PEB-derived MSCs, with a 4.9-fold greater area covered by Oil Red O stained lipid vesicles at the end of the assay (Figure 1B).

With respect to in vitro osteogenesis, day 21 calcium deposition by differentiated PEB-derived MSCs was 3-fold greater compared to matched EST-derived MSCs (*p* < 0.05, Figure 1C). Given the known reciprocal relationship between MSC pre-osteogenic and pre-adipogenic commitment [40,41], these data indicate that EST and PEB MSCs are preferentially adipo- and osteogenically-committed, respectively. In chondrogenic differentiation conditions, the GAG contents of PEB- and EST-derived MSCs were comparable (Figure 1D).

Culture expanded cells from the PEB and EST met the minimal ISCT criteria for MSCs [33]. Both populations had a positive expression of CD73, CD90, and CD105 cells and lacked expression of CD14, CD19, CD34, CD45, and HLA-DR (Figure 1E). One EST sample displayed some CD34 expression, as reported for CD34 in soft tissue MSCs, including adipose tissue [42].

Baseline gene expression data in undifferentiated MSCs supported the functional data and also showed significant differences relating to transcripts characteristic for lineage differentiation and stromal function (Supplementary Figure S1). Runt-related transcription factor 2 (*RUNX2*) was expressed at slightly higher levels by undifferentiated PEB MSCs compared to matched EST MSCS, though this was not statistically significant. Supporting the higher osteogenic potential of PEB MSCs compared to EST MSCs was the Dickkopf-related protein 1 (*DKK-1*), which had a higher expression in EST MSCs than matched PEB MSCs. The expression of *DKK-1* has been linked to the inhibition of osteogenesis, whilst also playing a role in adipogenesis [43,44]. In agreement with functional data, peroxisome proliferator-activated receptor gamma (*PPARγ*) had a significantly higher basal expression in EST MSCs compared to matched PEB MSCs (*p* < 0.05 *PPARγ*). Stromal cell-derived factor-1 (*CXCL12*) had a higher basal expression in EST MSCs compared to matched PEB MSCs (*p* < 0.05), while CCAAT Enhancer Binding Protein Beta (*CEBPβ*) had a higher expression in PEB MSCs. Interestingly, though both *IL-17RC* and TNFRS1A (*TNFR1*) were similarly expressed in PEB MSCs and EST MSCs, *IL-17RA* had a significantly higher basal expression in PEB MSCs compared to matched EST MSCs (*p* < 0.05).

### 3.2. IL-17A or TNF Inhibition of PEB- and EST-Derived MSC Adipogenesis

**Figure 2:**
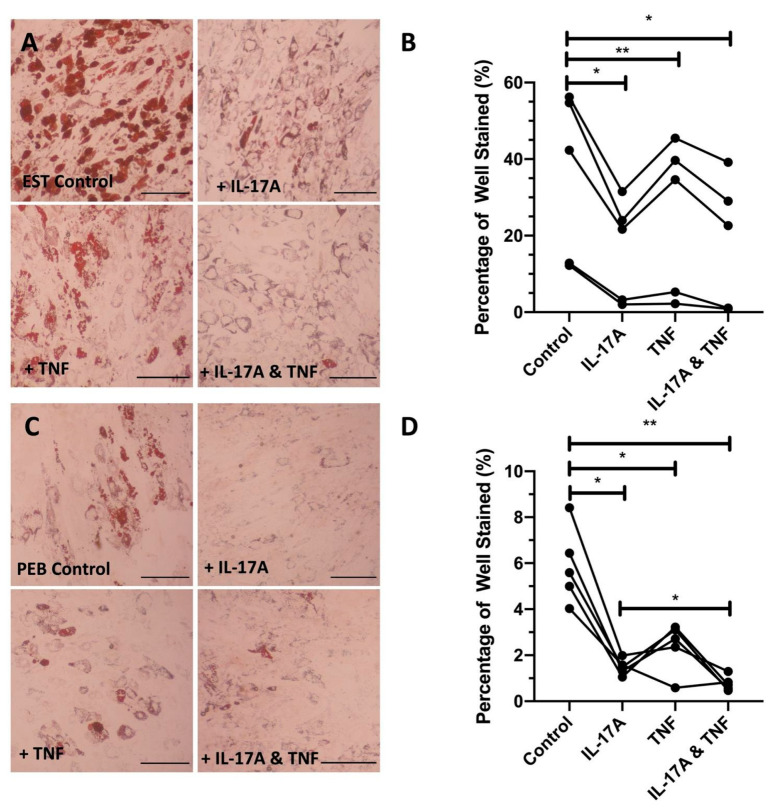
Representative images showing the cytokine inhibition of adipogenesis in both entheseal soft tissue (EST) (**A**) and peri-entheseal bone (PEB) (**C**) mesenchymal stem cells (MSCs). Quantified significant reductions in adipogenesis by IL-17A, tumout necrosis factor (TNF), and co-stimulation with both IL-17A and TNF in both EST ((**B**), RM One-Way ANOVA with Tukey Post-Hoc Test, *n* = 5) and PEB ((**D**), RM One-Way ANOVA with Tukey Post-Hoc Test, *n* = 5) MSCs. IL-17A used at 50 ng/mL and TNF at 1 ng/mL. * = *p* < 0.05, ** = *p* < 0.01. Scale bar = 250 µm.

The adipogenesis of both PEB and EST MSCs treated with either IL-17A (*p* < 0.01), TNF (*p* < 0.01), or their combination (*p* < 0.01) was significantly decreased compared to complete adipogenic media without cytokine supplementation (Figure 2).

Compared to control adipogenic media, the addition of IL-17A caused lipid droplets to noticeably reduce in size (EST: 2.0-fold reduction and PEB: 3.8-fold reduction), indicating that early vesicle fusion was inhibited, and mature adipocyte formation was prevented, which is demonstrated by the suppression of associated adipogenic transcripts (Figure 2A,B). TNF on its own did not show the same levels of adipogenesis inhibition (1.2- and 2.1-fold change for EST and PEB, respectively, Figure 2B). Compared to control media, the combination of IL-17A and TNF showed a 1.9- and 7.9-fold decrease for EST and PEB MSCs, respectively, in lipid staining, though this was not seen to be significantly different from TNF alone. The combination of IL-17A and TNF in PEB MSCs further reduced adipogenesis compared to IL-17A alone (*p* < 0.05, Figure 2C,D). Overall, in both types of MSCs, IL-17A was a stronger inhibitor of adipogenesis compared to TNF and no strong synergistic effect was observed for the cytokine combination.

### 3.3. IL-17A Moderately Increases the Osteogenesis of MSC Populations from Both Sites

To account for possible subtle effects of FCS on PEB and EST MSC osteogenesis and given the fact that PEB MSCs were more osteogenic (Figure 1C), two FCS concentrations in the osteogenic media (5% and 10%) were used to investigate the effects of IL-17A and TNF on PEB and EST MSC osteogenesis.

**Figure 3:**
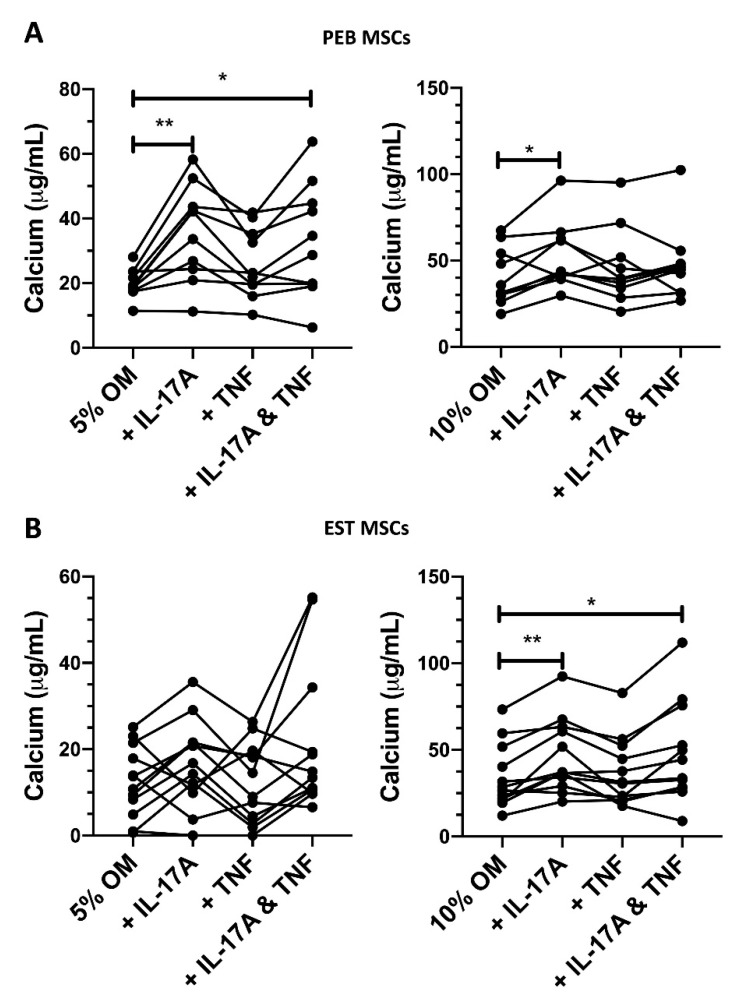
Changes in peri-entheseal bone (PEB) ((**A**), 5% FCS RM One-Way ANOVA with Tukey Post-Hoc Test *n* = 10, 10% FCS Friedman’s Test with Dunn’s Multiple Comparisons *n* = 10) and entheseal soft tissue (EST) ((**B**), 5% FCS Friedman’s Test with Dunn’s Multiple Comparisons, 10% FCS RM One-Way ANOVA with Tukey Post-Hoc Test *n* = 10) mesenchymal stem cell (MSC) osteogenesis following 14-day culture in osteogenic media containing cytokines and either 5% or 10% FCS. IL-17A used at 50 ng/mL and TNF at 1 ng/mL. * = *p* < 0.05, ** = *p* < 0.01. OM = osteogenic media.

In PEB MSCs cultured in 5% FCS osteogenic conditions, IL-17A induced a 2-fold increase in calcium production (Figure 3A, *p* < 0.01) and a 1.3-fold increase was seen for 10% FCS osteogenic conditions (Figure 3A, *p* < 0.05).

In 5% FCS osteogenic media, TNF induced a small non-significant increase in the osteogenesis of PEB MSCs (1.3-fold increase, Figure 3A). When using the stimulation of both IL-17A and TNF in 5% FCS osteogenic media, there was a significant 1.65-fold increase in calcium accumulation (*p* < 0.05).

EST-derived MSCs are generally poor at osteogenesis (Figure 1C), and this was seen in the amount of variability in calcium production in 5% FCS osteogenic media when stimulated by IL-17A (Figure 3B). When stimulating with IL-17A in 10% FCS osteogenic media, there was a modest 1.34-fold increase (*p* < 0.01, Figure 3B) in calcium accumulation. A combination stimulation of IL-17A and TNF in 10% FCS osteogenic media also showed a 1.32-fold increase (*p* < 0.05) in osteogenesis. Overall, in both types of MSCs, IL-17A was a stronger enhancer of osteogenesis compared to TNF and no strong synergistic effect was observed for the cytokine combination.

### 3.4. Adipogenic Transcripts Are Suppressed by IL-17A

**Figure 4:**
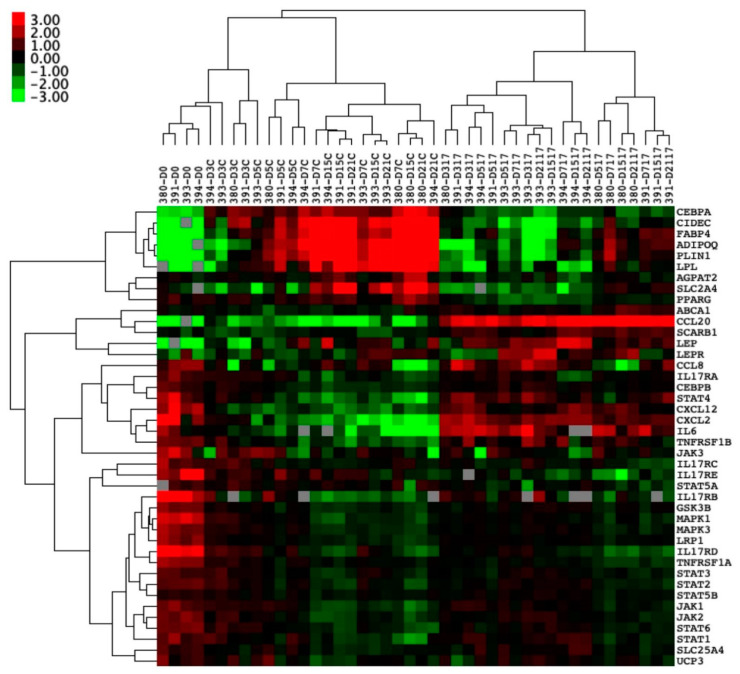
Entheseal soft tissue (EST) mesenchymal stem cells (MSCs) (*n* = 4) undergoing 21-day adipogenesis ± IL-17A. Hierarchical clustering demonstrating the distinct clustering of unstimulated MSCs undergoing adipogenesis away from MSCs stimulated by IL-17A. Colour denotes the relative expression of Hypoxanthine Phosphoribosyltransferase 1 (*HPRT1)*, with green representing low, black representing equal, red representing higher, and gray representing below detection. Numbers denote the sample ID, and D indicates the days in culture. IL-17A used at 50 ng/mL.

**Figure 5:**
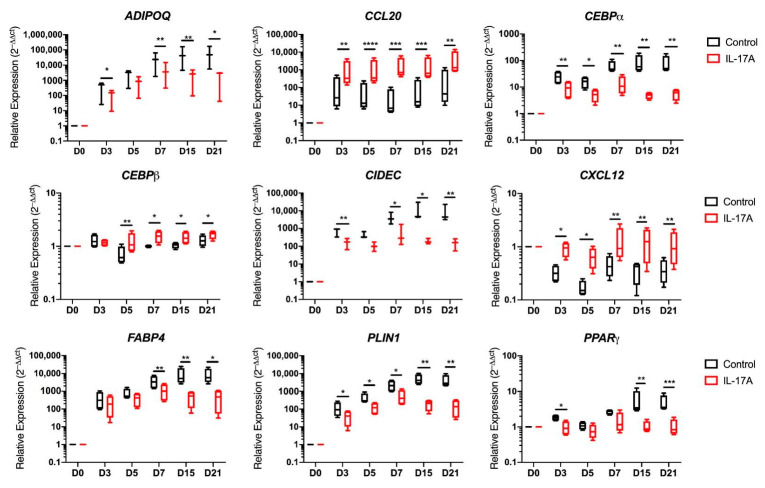
Entheseal soft tissue (EST) mesenchymal stem cells (MSCs) (*n* = 4) undergoing 21-day adipogenesis ± IL-17A (50 ng/mL). Transcripts relating to adipogenesis and vesicle fusion (paired *t*-tests) showing significant downregulation by the stimulation of IL-17A. Transcripts relating to MSC stromal support (*CXCL12*) and inflammatory cell migration (*CCL20*) showing significant upregulation by the stimulation of IL-17A compared to a control adipogenic media. * = *p* < 0.05, ** = *p* < 0.01, *** = *p* < 0.001, and **** = *p* < 0.0001.

Given that an inverse relationship existed between the augmentation of MSC osteogenesis and the simultaneous suppression of adipogenesis [40], we further investigated the basis for IL-17A’s impact on MSC adipogenesis and focused on adipogenic-related transcript expression (*n* = 4 donors) at five sequential time points over a 3-week differentiation period under IL-17A stimulation (Figure 4 and Figure 5).

Hierarchal clustering showed clear clusters with the downregulation of genes associated with adipogenesis when stimulated by IL-17A and genes associated with immune cell trafficking (Figure 4).

A significant suppression of transcripts relating to vesicle fusion and maturation, as well as to the adipogenic master regulator *PPARγ*, was evident (Figure 5) [45]. Another transcript that co-operates with *PPARγ* in mature adipocyte development was CCAAT Enhancer Binding Protein Alpha (*CEBPα*) [45], which was significantly downregulated by IL-17A stimulation from as early as Day 3 into differentiation (*p* < 0.01) and remained suppressed at every time point in the differentiation. The vesicle fusion proteins Perilipin-1 (*PLIN1*) and Cell Death Inducing DFFA like Effector C (*CIDEC*) were downregulated from day-3 onwards compared to control adipogenic media (*p* < 0.05 *PLIN1*, *p* < 0.01 *CIDEC*, Figure 5).

Typical transcripts associated with mature adipocytes were significantly downregulated, including Fatty Acid-Binding Protein 4 (*FABP4*) (*p* < 0.01) and Adiponectin (*ADIPOQ*) (*n* = 3, *p* < 0.01) from day 7 onwards in cultures exposed to IL-17A (Figure 5). IL-17A stimulation increased the expression of CCAAT Enhancer Binding Protein Beta (*CEBPβ*) (*p* < 0.01 at day 5, *p* < 0.05 from day 7–21) when compared to control adipogenic media, which is consistent with previous publications, showing that early adipogenic *CEBPβ* transcript elevation decreases in expression as adipogenesis matures by acting as an early *PPARγ* promoter [45].

The hierarchical clustering of PEB-derived MSC adipogenic genes did not produce as clear clusters (Supplementary Figure S2A), though the same inhibition by IL-17A was seen, consistent with staining for lipid vesicles. Notably, at day 21, *CIDEC* was significantly downregulated (*p* < 0.001) by exposure to IL-17A (Supplementary Figure S2B), as well as *FABP4* and *PLIN1* (*p* < 0.001 Supplementary Figure S2C,D), and this fits with the same pattern of inhibition seen in EST MSCs, despite the lower degree of initial expression.

Hierarchical cluster analysis also showed that a chemokine (*CXCL12*) characteristic for undifferentiated MSCs [46,47] remained elevated in adipogenic cultures exposed to IL-17A during adipogenesis. *CXCL12* in particular remained significantly elevated throughout the course of differentiation (Figure 5, *p* < 0.01). C-C Motif Chemokine Ligand 20 (*CCL20*) transcripts were also significantly upregulated by IL-17A stimulation from day 3 onwards (*p* < 0.01, Figure 5). CCL20 is known to play a role in SpA and acts as a T-cell and macrophage chemoattractant [19,22].

### 3.5. Undifferentiated EST and PEB MSCs Secrete CCL20 with IL-17A and TNF Co-Stimulation and CCR6+ T-Cells Are Present in the Enthesis

To investigate the functional significance of IL-17A and TNF stimulation with respect to MSC CCL20 secretion, we performed ELISA after 48 h stimulation on >90% confluent cultures. We also measured its sole receptor CCR6 on entheseal CD4+ and CD8+ cells, given that this chemokine axis is thought to be involved in IL-17A-driven inflammation at the enthesis [48].

**Figure 6:**
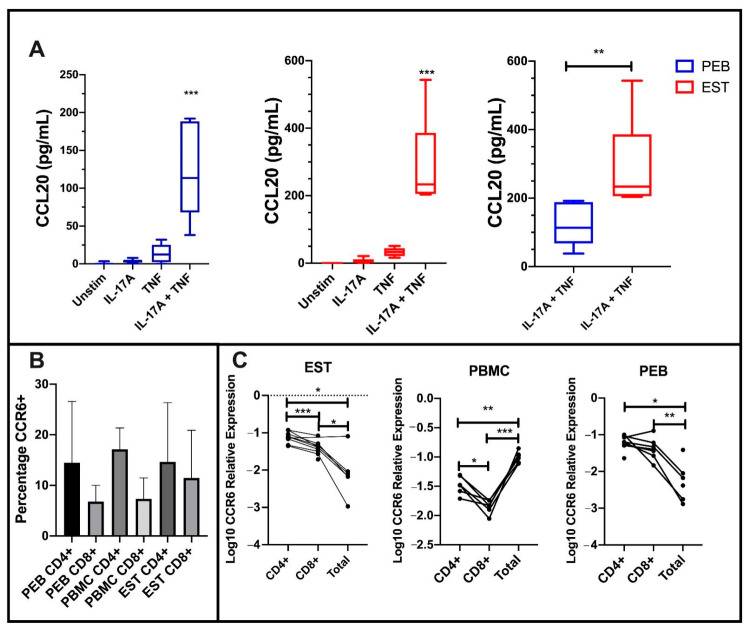
C-C Motif Chemokine Ligand 20 (CCL20) protein expression from stimulated mesenchymal stem cells (MSCs) and CCR6 expression on CD4 and CD8 T-cells at the enthesis. (**A**) Co-stimulation of both peri-entheseal bone (PEB) and entheseal soft tissue (EST) MSCs with IL-17A and TNF significantly increased CCL20 protein expression compared to the unstimulated control (Friedman’s Test with Dunn’s Multiple Comparisons, *n* = 6). EST MSCs secrete significantly more CCL20 than PEB MSCs (Mann–Whitney Test, *n* = 6). (**B**) CD4+ T-cells display a higher percentage expression compared to CD8+ T-cells in PEB, EST, and peripheral blood mononuclear cells (PBMC). (**C**) Transcript analysis shows higher levels of *CCR6* expression on CD4 cells compared to CD8 (RM One-Way ANOVA with Tukey Post-Hoc Test, PEB *n* = 8, EST *n* = 9, PBMC *n* = 6). Total represents all CD45+ cells from the tissue. IL-17A used at 100 ng/mL and TNF at 10 ng/mL. * = *p* < 0.05, ** = *p* < 0.01, and *** = *p* < 0.001.

Stimulation using either IL-17A or TNF alone did not significantly increase CCL20 protein secretion from either PEB- or EST-derived MSCs. However, following co-stimulation with IL-17A and TNF, there was a significant increase in CCL20 protein secretion from both PEB MSCs and EST-derived MSCs (both *p* < 0.001, Figure 6A).

As expected, EST-derived MSCs secreted significantly more CCL20 protein than PEB-derived MSCs (*p* < 0.01, Figure 6A). The surface CCR6 expression on CD4+ and CD8+ entheseal T-cells was similar to peripheral blood mononuclear cells (PBMCs) from the same patients (Figure 6B). At a transcript level, entheseal CD4+ and CD8+ T cells expressed higher levels of *CCR6* compared to all other entheseal cells. This was in contrast to the blood, where CD4+ and CD8+ cells showed a lower expression of *CCR6* when compared to all other leukocytes (Figure 6C). Taken together, these findings suggest a broad impact of IL-17A and TNF on the MSC function, including the tissue positioning of relevant cytokine-producing lymphocytes.

## 4. Discussion

Herein, we report the identification of two functionally distinct populations of MSCs at the normal human spinal enthesis. The PEB-derived MSCs had an intrinsically higher capability to make bone, probably in keeping with their native osseous topography, and the EST-derived MSCs surprisingly had a much higher adipogenic differentiation potential. TNF and IL-17A augmented MSCs’ osteogenic and CCL20 production, which could lead to immune cell recruitment. In line with the literature and the reciprocal link between MSC-mediated osteogenesis and -adipogenesis [40,41], these same cytokines and IL-17A in particular suppressed the adipogenic function. These findings indicate that disease-relevant cytokines to AS appear to be capable of “fine tuning” the MSC function in vitro.

The human spinal enthesis is an area subjected to continual loading throughout life [7,49]. Normal peripheral and axial entheseal tissues show microcracks in health, thus affording an opportunity for direct cellular communication between bone and soft tissue [7,49]. It is known that various entheseal attachments have plentiful adipocytes that likely act as shock absorbers at such stressed anchorage sites [3]. We identified two MSC populations in the PEB and EST that showed adipocyte differentiation and were thus capable of producing adipocytes acting as “space fillers” and joint stabilizers after damage or inflammation. Surprisingly, the enthesis MSC adipogenic potential was greater in the soft tissue than on the bone side. This is different from other ligamentous MSC populations, notably the anterior cruciate ligament, which has a worse adipogenic potential when compared to bone marrow MSCs from total knee arthroplasty [50]. However, our results are consistent with the low levels of adipocytes and CD34 staining seen in the EST, suggesting an enhanced adipogenic potential due to potentially adipogenically-committed MSCs. Our findings therefore point towards a potential role for MSC adipogenesis in spinal homeostasis and it is also noteworthy that adipogenesis is common in the fatty corners of AS and in degenerative arthritis [11].

Samples for experiments were selected randomly, with no bias for either sex or age of the sample. Post-experimental analysis showed no relationship for the effects of cytokines on MSCs for either the age or sex of the sample. However, during adipogenesis, older donor samples were noted as having a poorer adipogenic potential in control media, but importantly, their cytokine responses were identical to those of younger donor samples.

The SpA-associated cytokines IL-17A and TNF caused significant reductions in the adipogenic potential of both EST MSCs and PEB MSCs, with a greater impact of IL-17A adipocyte vesicle maturation suppression. *CIDEC* and *PLIN1*, which are both transcripts which enable the fusion of lipid vesicles to progress into more mature adipocytes [51], were seen to be significantly downregulated in EST-derived MSCs stimulated with IL-17A. This was supported by the significant downregulation of *PPARγ*, meaning that subsequent transcripts associated with mature adipocytes (*CEBPα*, *FABP4*, and *ADIPOQ*) [45] were further downregulated. Notably, *CXCL12* remains at levels similar to those of undifferentiated MSCs in cultures stimulated IL-17A, which suggests that IL-17A inhibits the differentiation of EST-derived MSCs into adipocytes, whilst maintaining their ability to attract immune-lineage cells. Though literature for IL-17A’s role in the formation of the fatty tissues is sparse, our findings are similar to those seen in investigations on obesity and the potential role of IL-17A in the development of insulin resistance [52,53]. IL-17A induced the downregulation of proteins associated with protection against lipolysis (*PLIN1* [54]). Our in vitro findings show that the IL-17A suppression of adipogenesis and augmentation of osteogenesis mirrors observations in AS where fat formation is a post-inflammatory phenomenon [11,55]. This is also in keeping with the known MSC biology, where full commitment to osteogenesis or adipogenesis represents two different fates [40].

We noted that, in addition to reducing in vitro adipogenesis and bolstering in vitro osteogenesis, IL-17A/TNF augmented both CCL20 transcript and protein production. CCL20 is a known chemoattractant for CCR6+ T-cells [22], with EST MSCs secreting CCL20 when stimulated by IL-17A and TNF, which are both known to play a role in the progression of AS [56]. Elevated CCL20 in response to SpA-associated inflammatory cytokines could lead to the recruitment of more Th17 T-cells, which are implicated in the pathogenesis of AS [20]. This lends credence to the idea of repeating waves of inflammation and IL-17A secretion, which, as mentioned above, could help resolve the ‘shiny corners’. In the SKG mouse model, which mirrors the SpA-like pathology, disease can be substantially reduced by blocking CCR6 [48]. We did not look at the *CCL20* transcript expression from MSC undergoing osteogenesis, due to primarily focusing on the early phases of AS, notably, the development of ‘shiny corners’ [11], and how these are potentially involved in the further recruitment of immune cells and subsequent inflammation leading to osteogenesis at the enthesis requires further study.

It is important to acknowledge the limitations associated with in vitro differentiation assays, with their reliance on corticosteroids for differentiation induction, and the difficulty in extrapolating the results to the in vivo scenario. Dexamethasone important in the initial stages of in vitro osteogenic differentiation is likely to play an inhibitory role in any inflammatory cytokine signaling. While some studies have reported that TNF increases MSCs’ osteogenic potential [26], we found that TNF only increased spinal MSC osteogenesis in PEB-derived MSCs in 5% FCS osteogenic media. Further work would be needed to fully determine why this is the case across these populations of MSCs, though these cells may require an additional stimulus to respond to TNF in an osteogenic manner. Dual stimulation with IL-17A and TNF significantly increased the osteogenic potential of both PEB and EST MSCs, matching the previously reported synergistic effects on the osteogenic potential of MSCs from bone marrow and synovium [26,27]. Consistently, the addition of IL-17A induced significant increases in the osteogenic potential of MSCs from both the PEB and EST MSCs. This finding fits with studies looking at other MSC sources, which showed increases in their osteogenic potential with IL-17A stimulation [12,15], though it should be noted that the literature is not unanimous in this finding [57,58]. Collectively, our findings indicate that TNF and IL-17A, although best known for inflammation modulation in AS, are capable of fine tuning the MSC function, even in health. This fine-tuning concept is supported by the presence of innate and adaptive immune lymphocytes capable of IL-17A production in health [18,19,20].

## 5. Conclusions

In conclusion, we have identified two functionally distinct MSC populations at the normal human spinal enthesis. These two populations both display an increased osteogenic potential following stimulation by IL-17A, with TNF only enhancing osteogenesis in PEB-derived MSCs, and these populations may regulate local lymphocyte populations capable of further cytokine production. Adipogenesis was significantly inhibited by the addition of IL-17A, with transcripts showing an increased expression of chemoattractant proteins. The CCL20 transcript expression correlated with a significantly increased protein expression when MSCs were stimulated with IL-17A/TNF, which in turn was significantly higher in EST MSCs than PEB MSCs. These findings provide novel insights into how SpA-associated cytokines that are successfully targeted therapeutically in SpA modulate the MSC function at the healthy enthesis. Indeed, we propose that this ability in “fine tuning” MSC activity in health may be dysregulated in disease, which requires further study.

## Data Availability

All data relevant to the study are included in the article or uploaded as supplementary information.

## Supplementary Materials

The following are available online at https://www.mdpi.com/2073-4409/10/2/341/s1: Figure S1: Gene transcript expression for transcripts relating to osteogenesis, adipogenesis, and stromal function in undifferentiated MSCs from both PEB and EST; Figure S2: Hierarchal clustering of PEB MSCs after adipogenesis with IL-17A stimulation; Table S1: Complete list of flow cytometry antibodies used; Table S2: Complete list of TaqMan Assays used for the analysis of gene expression; Table S3: List of samples used for each experiment.
