# Supplementary material for: IL-17A and TNF Modulate Normal Human Spinal Entheseal Bone and Soft Tissue Mesenchymal Stem Cell Osteogenesis, Adipogenesis, and Stromal Function
Source: Cells. 2021 Feb 6;10(2):341. doi: 10.3390/cells10020341 (PMC7915379; doi:10.3390/cells10020341)
Supplement: Supplementary file 1 [file cells-10-00341-s001.pdf]

cells MDPI

Supplementary Materials

Table S1. Complete list of flow cytometry antibodies used.

Target:	Clone:	Manufacturer:
CD3	UCHT1	BD Biosciences, San Jose, CA, USA
CD4	RPA-T4	BD Biosciences, San Jose, CA, USA
CD8	SK1	BD Biosciences, San Jose, CA, USA
CD14	REA599	Miltenyi Biotec, Bergisch Gladbach, Germany
CD19	HIB19	BD Biosciences, San Jose, CA, USA
CD34	563	BD Biosciences, San Jose, CA, USA
CD45	HI30	BD Biosciences, San Jose, CA, USA
CD73	AD2	Miltenyi Biotec, Bergisch Gladbach, Germany
CD90	F15-42-1	Bio-rad laboratories, Watford, UK
CD105	REA794	Miltenyi Biotec, Bergisch Gladbach, Germany
HLA-DR	L243	BD Biosciences, San Jose, CA, USA
CCR6	11A9	BD Biosciences, San Jose, CA, USA

Table S2. Complete list of TaqMan Assays used for analysis of gene expression.

Gene:	Gene Name:	Assay ID:
ABCA1	ATP binding cassette subfamily A member 1	Hs01059137_m1
ABCG1	ATP binding cassette subfamily G member 1	Hs00245154_m1
SLC25A4	Solute carrier family 25 member 4	Hs00154037_m1
SLC2A4	Solute carrier family 2 member 4	Hs00168966_m1
GSK3B	Glycogen synthase kinase 4 beta	Hs00275656_m1
SCARB1	Scavenger receptor class B receptor 1	Hs00969821_m1
LRP1	LDL receptor related protein 1	Hs00233856_m1
LEP	Leptin	Hs00174877_m1
LEPR	Leptin receptor	Hs00174492_m1
LPL	Lipoprotein lipase	Hs00173425_m1
ADIPOQ	Adiponectin	Hs00605917_m1
AGPAT2	1-acylglycerol-3-phosphate O-acyltransferase 2	Hs00944961_m1
FABP4	Fatty acid binding protein 4	Hs00609791_m1
PPARG	Peroxisome proliferator activated receptor gamma	Hs01115513_m1
CEBPA	CCAAT/enhancer binding protein alpha	Hs00269972_s1
CEBPB	CCAAT/enhancer binding protein beta	Hs00942496_s1
PREF1	Pre-adipocyte factor 1	Hs00171584_m1
PLIN1	Perilipin 1	Hs00160173_m1
PLIN5	Perilipin 5	Hs00965990_m1
CIDEA	Cell death inducing DFFA like effector A	Hs00154455_m1
CIDEC	Cell death inducing DFFA like effector C	Hs00535724_gH
UCP3	Uncoupling protein 3	Hs01106052_m1
CCL8	C-C motif chemokine ligand 8	Hs04187715_m1
CCL20	C-C motif chemokine ligand 20	Hs01011368_m1
CXCL2	C-X-C motif chemokine ligand 2	Hs00601975_m1
CXCL12	C-X-C motif chemokine ligand 12	Hs00171022_m1
IL6	Interleukin 6	Hs00174131_m1
MAPK3	Mitogen-activated protein kinase 3	Hs00385075_m1
MAPK1	Mitogen-activated protein kinase 1	Hs01046839_m1

JAK1	Janus kinase 1	Hs01026983_m1
JAK2	Janus kinase 2	Hs01078136_m1
JAK3	Janus kinase 3	Hs00169663_m1
STAT1	Signal transducer and activator of transcription 1	Hs01013996_m1
STAT2	Signal transducer and activator of transcription 2	Hs01013123_m1
STAT3	Signal transducer and activator of transcription 3	Hs00374280_m1
STAT4	Signal transducer and activator of transcription 4	Hs01028017_m1
STAT5A	Signal transducer and activator of transcription 5A	Hs00234181_m1
STAT5B	Signal transducer and activator of transcription 5B	Hs00273500_m1
STAT6	Signal transducer and activator of transcription 6	Hs00598625_m1
IL17RA	Interleukin 17 receptor A	Hs01056316_m1
IL17RB	Interleukin 17 receptor B	Hs00218889_m1
IL17RC	Interleukin 17 receptor C	Hs00994305_m1
IL17RD	Interleukin 17 receptor D	Hs00296982_m1
IL17RE	Interleukin 17 receptor E	Hs00979824_m1
TNF	Tumour necrosis factor	Hs99999043_m1
TNFRSF1A	TNF Receptor Superfamily Member 1A	Hs01042313_m1
TNFRSF1B	TNF Receptor Superfamily Member 1B	Hs00153550_m1
CCR6	C-C motif chemokine receptor 6	Hs00171121_m1
HPRT	Hypoxanthine phosphoribosyltransferase 1	Hs99999909_m1
DKK-1	Dickkopf-Related Protein 1	Hs00183740_m1
RUNX2	Runt-related transcription factor 2	Hs00231692_m1

Table S3. List of samples used for each experiment.

Sample ID:	Age:	Sex:	Application:
RC121	69	Male	Chondrogenesis, Adipogenesis
RC123	83	Male	Chondrogenesis, Adipogenesis
RC143	43	Male	Flow Cytometry
RC151	66	Female	Flow Cytometry
RC152	24	Female	Flow Cytometry
RC153	68	Female	Flow Cytometry
RC154	78	Male	Flow Cytometry
RC160	56	Female	Flow Cytometry
RC183	69	Male	Flow Cytometry
RC184	47	Male	Flow Cytometry
RC210	71	Male	Flow Cytometry
RC211	80	Female	Flow Cytometry
RC212	18	Female	Colony Forming Unit (CFU), Flow Cytometry
RC217	66	Female	Osteogenesis, Chondrogenesis, Adipogenesis
RC219	68	Male	Osteogenesis, CFU, Osteogenesis + Cytokines
RC220	75	Female	CFU
RC221	80	Female	Osteogenesis, CFU, Flow Cytometry, Osteogenesis + Cytokines
RC222	12	Female	Adipogenesis + Cytokines, Osteogenesis + Cytokines
RC223	19	Female	Osteogenesis, Adipogenesis + Cytokines
RC224	13	Female	Osteogenesis, CFU, Chondrogenesis, Adipogenesis
RC229	12	Female	CFU, Adipogenesis + Cytokines
RC232	68	Female	Flow Cytometry
RC234	20	Female	Osteogenesis, CFU, Osteogenesis + Cytokines
RC241	15	Male	CFU

RC242	23	Male	CFU, Osteogenesis + Cytokines
RC250	68	Female	Osteogenesis, CFU, Flow Cytometry
RC265	67	Male	Osteogenesis, CFU, Flow Cytometry, Adipogenesis, Osteogenesis + Cytokines
RC300	14	Female	Osteogenesis + Cytokines
RC303	83	Male	Osteogenesis, CFU, Osteogenesis + Cytokines
RC311	49	Male	CFU
RC312	13	Male	CFU
RC313	63	Male	CFU, Adipogenesis, Osteogenesis + Cytokines
RC317	66	Male	Osteogenesis, Adipogenesis + Cytokines, Osteogenesis + Cytokines
RC359	64	Female	Adipogenesis + Cytokines, Osteogenesis + Cytokines
RC380	15	Female	CFU, Adipogenesis Time Course, Osteogenesis + Cytokines, Flow Cytometry
RC391	14	Female	Adipogenesis Time Course, Osteogenesis + Cytokines,
RC393	53	Female	Adipogenesis Time Course, Osteogenesis + Cytokines,
RC394	74	Male	Adipogenesis Time Course

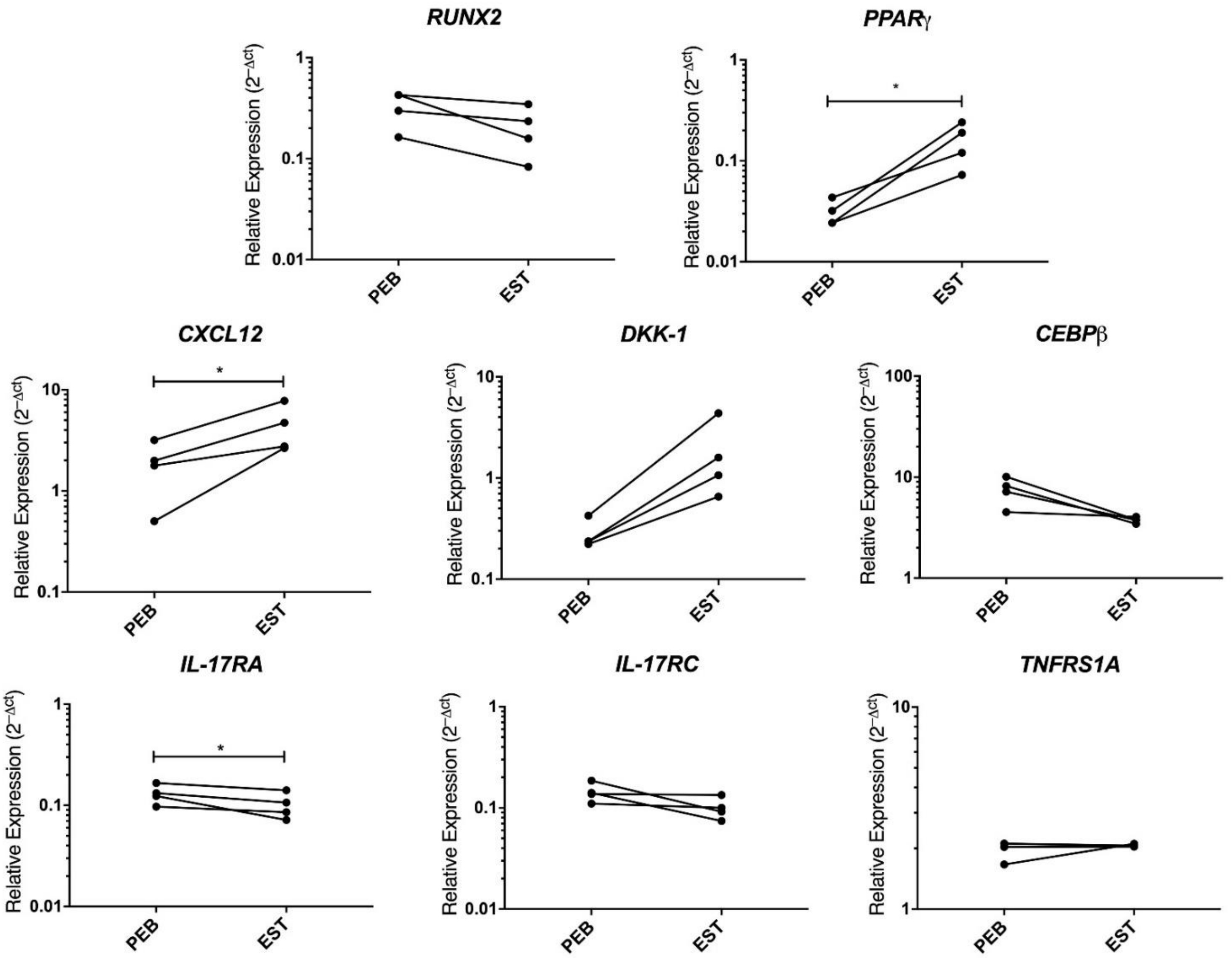

Figure S1. Gene transcript expression for transcripts relating to osteogenesis, adipogenesis and stromal function in undifferentiated mesenchymal stem cells (MSCs) from both peri-entheseal bone (PEB) and entheseal soft tissue (EST) ($n = 4$). EST MSCs showed significantly higher expression of peroxisome proliferator-activated receptor gamma (*PPARγ)* ($p < 0.05$) and Stromal cell-derived factor-1 (*CXCL12)* ($p < 0.05$). With transcripts associated with enhanced osteogenesis in Runt-related transcription factor 2 (*RUNX2*) being more expressed in PEB MSCs compared to match EST MSCs, though this was not significant. * = $p < 0.05$.

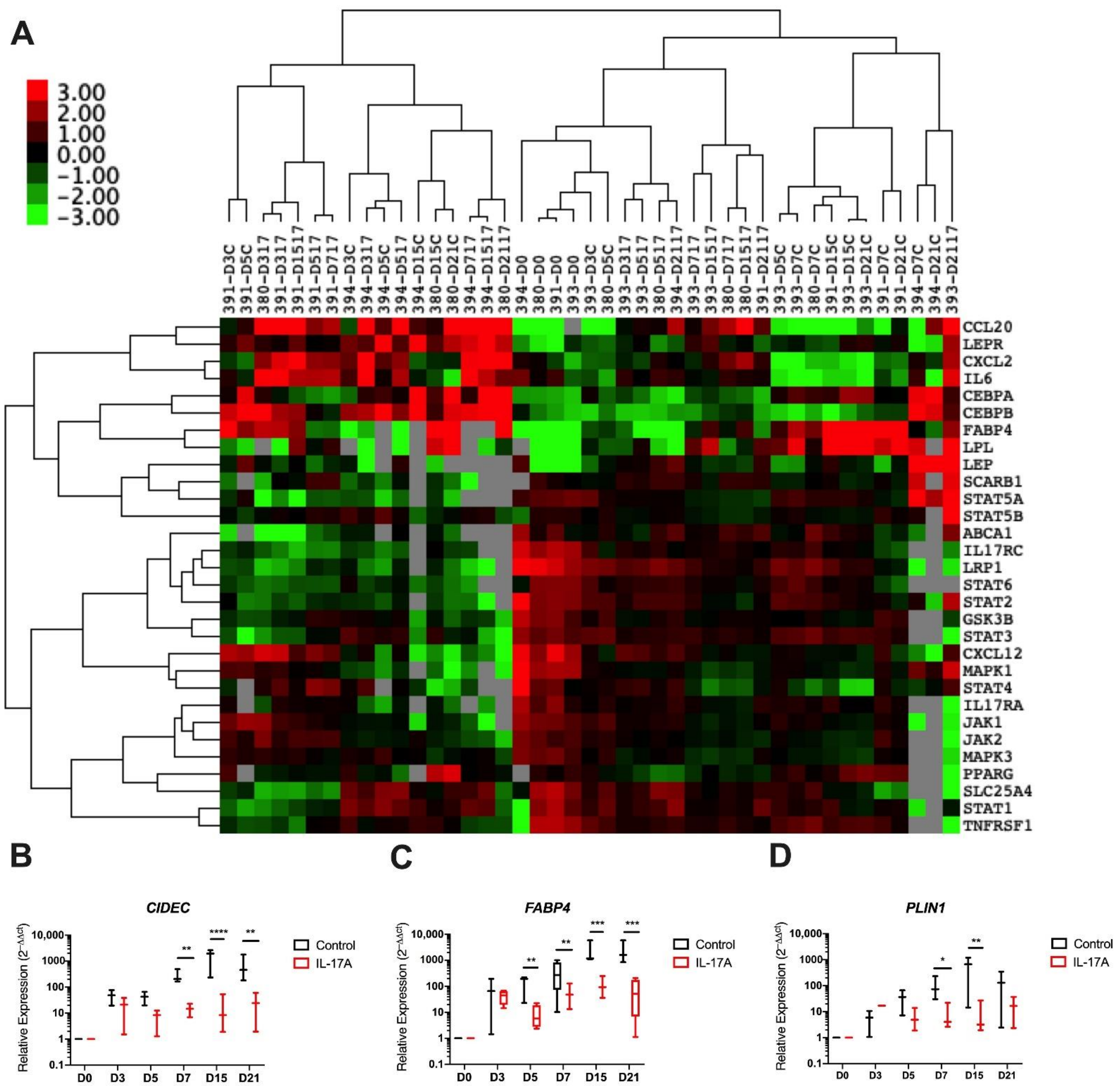

Figure S2. (**A**) Hierarchical clustering of peri-entheseal bone (PEB) mesenchymal stem cells (MSCs) is absent due to Table 75. of total values were detectable. Colour denotes relative expression to Hypoxanthine Phosphoribosyltransferase 1 (*HPRT1*), green-low, black-equal, red-higher, grey-below detection. Numbers denote sample ID, D-days in culture though fusion proteins Cell Death Inducing DFFA like Effector C (*CIDEC*) (**B**) and Perilipin-1 (*Plin1*) (**D**) all showed significant downregulating at various time points when stimulated by IL-17A. This was also true for Fatty Acid-Binding Protein 4 (*FABP4*) (**C**) showing downregulation by IL-17A from day 5 of PEB adipogenesis. * = $p < 0.05$, ** = $p < 0.01$, *** = $p < 0.001$, **** = $p < 0.0001$.
